# A High-Density Genetic Linkage Map and QTL Fine Mapping for Body Weight in Crucian Carp (*Carassius auratus*) Using 2b-RAD Sequencing

**DOI:** 10.1534/g3.117.041376

**Published:** 2017-06-08

**Authors:** Haiyang Liu, Beide Fu, Meixia Pang, Xiu Feng, Xiaomu Yu, Jingou Tong

**Affiliations:** * State Key Laboratory of Freshwater Ecology and Biotechnology, Institute of Hydrobiology, Chinese Academy of Sciences, Wuhan 430072, China; † Graduate School, University of Chinese Academy of Sciences, Beijing 100039, China

**Keywords:** crucian carp, SNP, genetic linkage map, QTL, comparative genomics, candidate growth gene

## Abstract

A high-resolution genetic linkage map is essential for a wide range of genetics and genomics studies such as comparative genomics analysis and QTL fine mapping. Crucian carp (*Carassius auratus*) is widely distributed in Eurasia, and is an important aquaculture fish worldwide. In this study, a high-density genetic linkage map was constructed for crucian carp using 2b-RAD technology. The consensus map contains 8487 SNP markers, assigning to 50 linkage groups (LGs) and spanning 3762.88 cM, with an average marker interval of 0.44 cM and genome coverage of 98.8%. The female map had 4410 SNPs, and spanned 3500.42 cM (0.79 cM/marker), while the male map had 4625 SNPs and spanned 3346.33 cM (0.72 cM/marker). The average recombination ratio of female to male was 2.13:1, and significant male-biased recombination suppressions were observed in LG47 and LG49. Comparative genomics analysis revealed a clear 2:1 syntenic relationship between crucian carp LGs and chromosomes of zebrafish and grass carp, and a 1:1 correspondence, but extensive chromosomal rearrangement, between crucian carp and common carp, providing evidence that crucian carp has experienced a fourth round of whole genome duplication (4R-WGD). Eight chromosome-wide QTL for body weight at 2 months after hatch were detected on five LGs, explaining 10.1–13.2% of the phenotypic variations. Potential candidate growth-related genes, such as an EGF-like domain and TGF-β, were identified within the QTL intervals. This high-density genetic map and QTL analysis supplies a basis for genome evolutionary studies in cyprinid fishes, genome assembly, and QTL fine mapping for complex traits in crucian carp.

Linkage mapping is an important area of genetic research, and high-density linkage maps provide a basic tool to gain insight into the genetic architecture of traits of evolutionary and economic interest (Naruse *et al.* 2000; Knapik *et al.* 1998). A high-density map can provide a lot of genetic information, such as chromosome structure, segregation distortion region, recombination rate, and recombination hotspots (Myers *et al.* 2005; Zhu *et al.* 2014; Guo *et al.* 2013; Shifman *et al.* 2006). Furthermore, it is an indispensable tool for fine-scale mapping of phenotypic traits of interest, candidate gene cloning, gene-centromere mapping, comparative genomic analysis, and genome assembly (Zhu *et al.* 2013, 2015; Peng *et al.* 2016; Xiao *et al.* 2015; Z. Wang *et al.* 2015; Tian *et al.* 2015; Shao *et al.* 2015; Li *et al.* 2015; Feng *et al.* 2015). SNPs are the most abundant genetic markers in the genomes of organisms, and have become the preferred markers for high-density map construction (Baird *et al.* 2008). The development of next-generation sequencing (NGS) technology provides the capacity for developing and genotyping large numbers of SNP markers rapidly and cost-effectively (Davey *et al.* 2011). Taking advantage of NGS, high-density linkage maps have been constructed using thousands of SNP markers in many aquaculture species, such as Atlantic salmon (Tsai *et al.* 2016), channel catfish (Li *et al.* 2015), Japanese flounder (Shao *et al.* 2015), Asian sea bass (Y. Wang *et al.* 2015), large yellow croaker (Xiao *et al.* 2015), Mexican tetra (Carlson *et al.* 2015), and oyster (Wang *et al.* 2016). Recently, a variety of genotyping-by-sequencing (GBS) methods have been developed for SNP discovery and genotyping, such as RAD (Baird *et al.* 2008), ddRAD (Peterson *et al.* 2012), GGRS (Chen *et al.* 2013), SLAF (Sun *et al.* 2013), and 2b-RAD (S. Wang *et al.* 2012). Among these methods, 2b-RAD is a simple and flexible method invented by S. Wang *et al.* (2012) that adopts type IIB restriction enzymes for genome-wide genotyping. Compared with other GBS methods, it allows screening of almost every restriction site in the genome, regulates genome coverage more flexibly, and has a simpler protocol for library preparation (S. Wang *et al.* 2012). 2b-RAD has been used successfully for constructing high-density maps and QTL fine mapping in several aquaculture species (Jiao *et al.* 2014; Shi *et al.* 2014; Cui *et al.* 2015; Tian *et al.* 2015; Dou *et al.* 2016; Fu *et al.* 2016; Palaiokostas *et al.* 2016).

Teleosts have undergone the third round (3R) of whole genome duplication (WGD) ∼350 million years ago (MYA), which is an additional round that mammals do not have (Jaillon *et al.* 2004; Meyer and Van de Peer 2005). The 3R-WGD may have facilitated the evolutionary innovations, and led to the diversity of teleosts (Taylor *et al.* 2003; Venkatesh 2003; Ravi and Venkatesh 2008). Recently, a variety of evidence (*e.g.*, chromosome numbers, genome size, orthologous genes, and comparative genomes) indicates that some fish species have experienced a fourth round (4R) of WGD, such as some fishes of salmonids (Berthelot *et al.* 2014; Lien *et al.* 2016; Danzmann *et al.* 2008; Guyomard *et al.* 2012; Timusk *et al.* 2011; Brieuc *et al.* 2014; Allendorf and Thorgaard 1984), catastomids (Uyeno and Smith 1972; Zhou *et al.* 2002), Cobitidae (Ferris and Whitt 1977; Kusunoki *et al.* 1994; Kitagawa *et al.* 2003), and Cyprininae (J.T. Wang *et al.* 2012; David *et al.* 2003; Xu *et al.* 2014; Larhammar and Risinger 1994; Risinger and Larhammar 1993; Ohno *et al.* 1967; Zhang *et al.* 2013; Zhao *et al.* 2013; Henkel *et al.* 2012). The 4R-WGD provides an excellent opportunity for studying evolutionary patterns of vertebrate genomes after an autotetraploid WGD (Davidson *et al.* 2010; Lien *et al.* 2016). Comparative genome analysis based on whole-genome sequencing (WGS) have provided unambiguous evidence for WGD in some fishes (Berthelot *et al.* 2014; Lien *et al.* 2016; Xu *et al.* 2014; Glasauer and Neuhauss 2014). Comparative genomics using linkage maps is an alternative approach to investigating chromosome reduplication and chromosomal rearrangements after WGD events in nonmodel fish (Zhang *et al.* 2013; Zhao *et al.* 2013; Guyomard *et al.* 2012; Brieuc *et al.* 2014; Gharbi *et al.* 2006). For example, a clear 2:1 relationship of common carp linkage groups and zebrafish (*Danio rerio*) chromosomes revealed an additional genome reduplication (Zhang *et al.* 2013; Zhao *et al.* 2013), and comparative mapping studies between salmonids showed extensive chromosomal rearrangements and differentiation (Guyomard *et al.* 2012; Brieuc *et al.* 2014; Gharbi *et al.* 2006; Naish *et al.* 2013).

Crucian carp (*Carassius auratus*) is widely distributed in Eurasia and is one of the most important freshwater fish species for Chinese aquaculture. Previous karyotype studies demonstrated that crucian carp has a diploid chromosome number of 2*n* = 100, a number twice as much most of other cyprinid fishes (2*n* = 50 or 48) (Knytl *et al.* 2013; Ojima *et al.* 1979; Kobayasi *et al.* 1970). Recent studies have revealed diploid (2*n* = 100), triploid (3*n* = 150), and tetraploid (4*n* = 200) crucian carp in natural populations, and these three ploidy populations often coexist with each other in natural waters (Liu *et al.* 2001; Xiao *et al.* 2011; Gui and Zhou 2010). Thereby, it was believed that crucian carp may have experienced multiple successive rounds of chromosome doubling, which provided an excellent material for genome duplication and evolution studies (Zhang *et al.* 2015; Gui and Zhou 2010; Zhou *et al.* 2002). Moreover, as an important farmed fish, the global aquaculture production of crucian carp reached 2.91 million tons in 2015 (FAO, 2016). Growth rate is one of the most important traits for breeding programs in crucian carp (Zhou *et al.* 2000a; Hulata 1995). Several strains of triploid crucian carp have been developed in China via gynogenesis or interspecific hybridization between crucian carp and common carp, which has allowed great progress in breeding, and significantly increased the production of crucian carp (Gui and Zhou 2010; Zhou *et al.* 2000a,b, 2001; Liu *et al.* 2001, 2004; Hulata 1995). However, the genetic basis and architecture for growth modulation in crucian carp are still poorly understood because few genetic and genomic resources are available (Liao *et al.* 2013). It is well known that growth is a typical quantitative trait controlled by multiple genes known as quantitative trait loci (QTL), and may be influenced by environmental factors (Lynch and Walsh 1998; Mackay 2001; Mackay *et al.* 2009). Traditional selective breeding methods have encountered some difficulties such as uncertainty, extensive workload, being time-consuming, and being slow to take effect. Hence, molecular breeding methods, such as marker assisted selection (MAS) and genomic selection, are needed to accelerate breeding process in fish (Yue 2014; Liu and Cordes 2004; Tong and Sun 2015).

The objectives of this study include: (i) construction of a high-density SNP-based linkage map using 2b-RAD technology in diploid crucian carp; (ii) comparative genomics analysis between crucian carp and zebrafish (*D. rerio*), grass carp (*Ctenopharyngodon idellus*), and common carp (*Cyprinus carpio*). (iii) QTL fine mapping for body weight, and identification of candidate genes that may involve early growth of crucian carp. This study will provide a framework for further studies on genome evolution, comparative genomics, and fine-scale QTL mapping for economic traits in crucian carp.

## Materials and Methods

### Mapping family and DNA extraction

A total of 102 adult crucian carp was collected from a wild population in the Zhangdu Lake (Wuhan, China) and used as candidate broodfish in the production of mapping families. Genetic distances among these fish were evaluated using 20 previously reported microsatellite loci (Zheng *et al.* 2010). In April 2015, 17 candidate F1 full-sib families were established by crossing 14 sires and 17 dams via artificial fertilization. Larval fish of each family were raised in a plastic tank, and fed with brine shrimp (nauplii of the Artemia) twice a day. Body weights were measured and recorded for all families at the age of 2 months posthatch. One family with the highest genetic distance and largest within-family phenotypic variations in body weight was selected as the mapping panel for linkage map construction and QTL analysis for early growth in this study. A total of 160 progenies was randomly selected from this family, and a small piece of clipped fin was sampled from both parents and progenies and stored in 95% ethanol for DNA extraction. Genomic DNA was extracted from preserved fin tissues following a standard phenol-chloroform DNA extraction procedure (Sambrook and Russell 2001). The quality of DNA was checked by a NanoDrop 2000 spectrophotometer (Thermo Scientific), and 1% agarose gel electrophoresis. The concentrations of all DNA samples were adjusted to 50 ng/μl. All experimental animal programs involved in this study were approved by the Animal Care and Use Committee at the Institute of Hydrobiology, Chinese Academy of Sciences.

### 2b-RAD library construction and de novo genotyping

Before library preparation, the number of possible restriction sites was calculated based on crucian carp genome draft assembly (unpublished data). The 2b-RAD library was prepared following the protocol originally described by S. Wang *et al.* (2012) with minor modifications (Fu *et al.* 2016). Two parents and 160 offspring were used for the construction of 2b-RAD libraries. Briefly, 250 ng of genomic DNA was digested with 1 unit *Bcg*I restriction enzyme (New England Biolabs) at 37° for 4 hr. The digested DNA was ligated at 16° for 8 hr with a 25 μl total volume reaction consisting of 1 unit T4 DNA ligase (New England Biolabs), 0.5 μM adapter 1 and adapter 2, 0.5 mM ATP (New England Biolabs). The ligation fragments were amplified in a 25 μl total volume consisting of 5 μl of ligated DNA, 0.5 μM P5 and P7 primer, 0.5 μM P4 and P6-BC primer, 0.75 mM dNTP (Shanghai Sangon, China), 5 μl 5× Phusion HF buffer, and 0.5 unit Phusion High-Fidelity DNA Polymerase (Thermo Scientific). The cycling conditions were: 98° for 30 sec; 98° for 20 sec, 63° for 50 sec, 72° for 30 sec for 15 cycles, 72° for 5 min. The amplification products were purified via retrieval from 8% polyacrylamide gels. All libraries were pooled with equal amount of products from each library to make a final library which was sequenced on a lane at the HiSeq2500 platform (Illumina). The raw read data were archived at the NCBI Sequence Read Archive (SRA) under accession number PRJNA327320.

Raw reads were first trimmed to remove adapter sequences, and the terminal 2-bp positions. Reads without restriction sites or containing long homopolymers (>10 bp), ambiguous bases (N), low-quality sequences (more than five positions with a quality <20) or mitochondrial origins were removed. The remaining trimmed reads with 32 bp in length were used for subsequent analysis. Filtered reads were analyzed with the software RADtyping program v1.0 (Fu *et al.* 2013) for *de novo* 2b-RAD genotyping. This software used stringent criteria in filtering candidate markers, and only those loci with at least four reads supporting were kept in the following analysis.

### Linkage map construction

Only those markers that segregated in parents and could be genotyped in at least 80% of the offspring were used for further analysis. Markers that present significant segregation distortion in the χ^2^ goodness-of-fit tests (*P* < 0.05) were eliminated in the linkage analysis. Slightly distorted markers (*P* > 0.05) were also used for linkage analysis. A consensus linkage map was constructed by JoinMap 4.1 program (Van Ooijen 2006) with a threshold LOD score of 15.0. Male- and female-specific linkage map calculations were performed using the function of “Create Maternal and Paternal Population Nodes” in JoinMap 4.1, with a threshold LOD score of 10.0. The visualized linkage maps were drawn using MapChart v2.2 (Voorrips 2002). The linkage groups (LGs) of crucian carp were named according to their homologous groups of common carp and zebrafish based on the results of comparative genome analyses. The expected genetic map length was calculated in two ways: *G*_e1_ (Fishman *et al.* 2001) and *G*_e2_ (Chakravarti *et al.* 1991), and the average of these two indexes was used as the predicted total genetic map length (*G*_e_). The recombination ratio of female to male was calculated by the ratios of mean distances of each LG in the female and male maps.

### Comparative genome analysis

All mapped 2b-RAD sequences (32 bp) were first aligned against the crucian carp genome draft assembly (unpublished data). Those markers mapped at a single genome position were then extended by adding 100 nucleotide sequences from each side. A total of 5734 extended 2b-RAD sequences was searched against the genomes of zebrafish (*Danio rerio*, GRCz10), grass carp (*Ctenopharyngodon idella*), and common carp (*C. carpio*) using the basic local alignment search tool (BLASTN) with an *e*-value cutoff of 1e−10. If a single marker sequence aligned multiple targets at different positions, only the top hit (lowest *e*-value) alignment was retained. The genomic synteny was visualized using the software Circos v0.67 (Krzywinski *et al.* 2009).

### QTL analysis for body weight

QTL mapping analysis was performed using the MapQTL 6.0 (Van Ooijen and Kyazma 2009) software program with a Multiple QTL Mapping (MQM) model. A mapping step size of 1 cM and five neighboring markers were used in QTL analysis. The genome-wide LOD threshold (significance level) or group-wide LOD threshold (suggestive level) were estimated using the permutation test (10,000 replicates) in MapQTL 6.0 with a confidence interval of 95%.

### Identification of potential candidate genes

Potential candidate genes within each QTL region were hunted through comparative genomics. We performed sequence similarity searches (BLASTN) for all QTL-associated SNP markers against the whole genome sequences of crucian carp (unpublished data) and common carp (Xu *et al.* 2014). Only annotated genes closest to the peak of corresponding QTL region were regarded as candidate genes.

### Data availability

The authors state that all data necessary for confirming the conclusions presented in the article are represented fully within the article.

## Results

### 2b-RAD genotyping

A total of 6.56, 3.98 and 177.44 million reads were produced by 2b-RAD sequencing for the female parents, and male parents and 160 progenies (1.11 million reads per progeny), respectively. After sequential quality filtering and sequence trimming, two parents’ reads were clustered into 124,367 representative reference tags, including 98,911 codominant tags (parent-shared), and 25,456 dominant tags (parent-specific). After removing the 4451 tags with insufficient sequencing depth, 96,328 codominant tags and 23,588 dominant tags were retained and used for constructing high-quality reference tags. Utilizing the constructed reference, a total of 14,732 polymorphic markers were detected, including 7310 codominant markers and 7422 dominant markers. After a Mendelian fit test (*P* > 0.05), and genotyping percentage (over 80% progenies) detection, 4073 codominant and 4693 dominant SNP markers were used to construct consensus map and sex-specific maps.

### High-density linkage map

**Figure 1:**
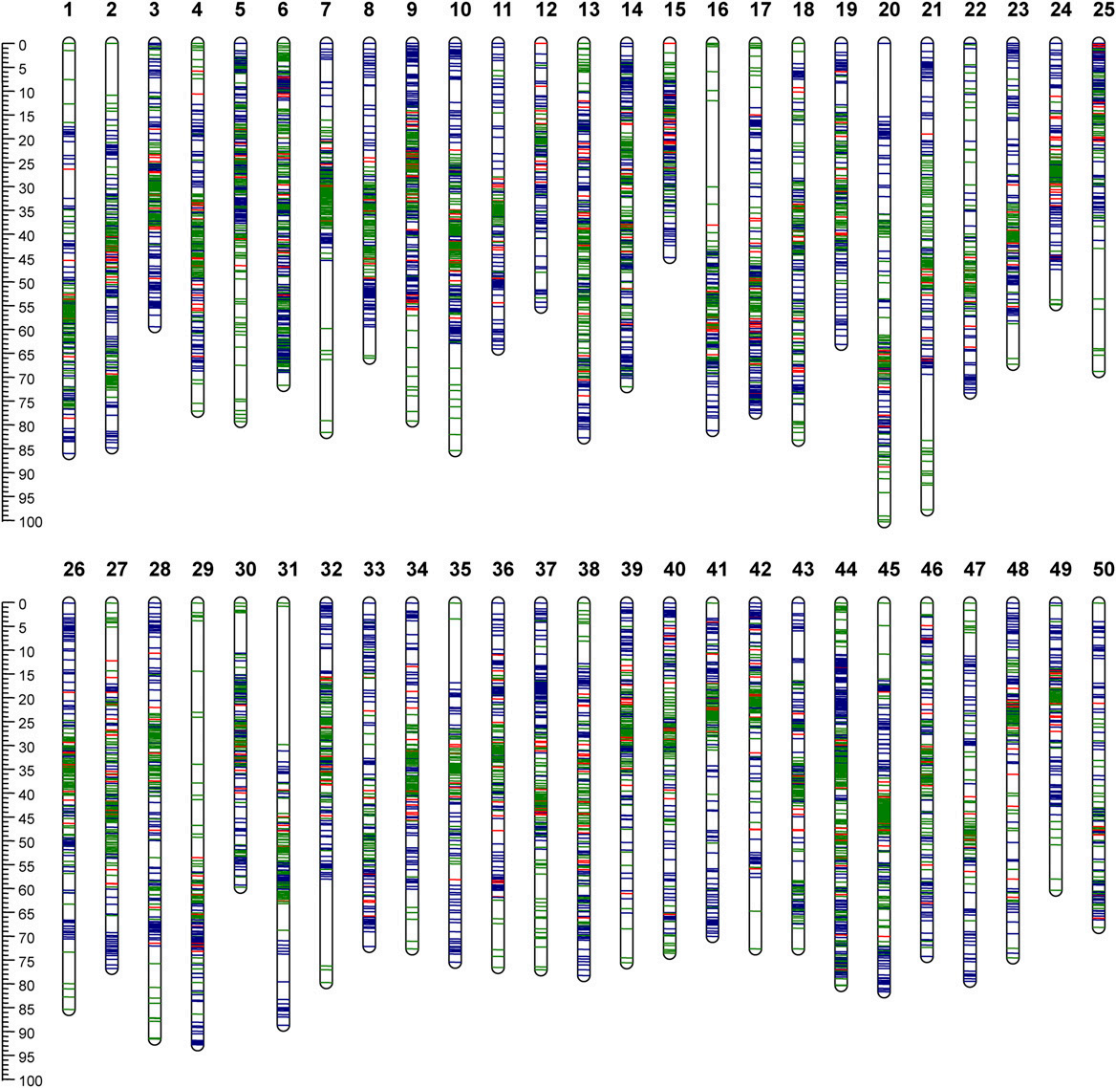
Genetic distances and marker distribution of 50 linkage groups in the consensus linkage map of crucian carp. Within each linkage group, blue, green, and red lines represent maternal heterozygous SNPs, paternal heterozygous SNPs, and SNPs heterozygous in both parents, respectively.

All polymorphic SNP markers were grouped into 50 linkage groups, which is consistent with the haploid chromosome number of crucian carp (2*n* = 100) (Knytl *et al.* 2013). The consensus map consisted of 8487 markers and spanned 3762.88 cM, with an average interval of 0.44 cM (Figure 1 and Table 1). The number of markers per group varied from 106 (LG 22 and LG 50) to 331 (LG 44), with an average of 169.74, and the length per group ranged from 44.95 cM (LG 15) to 100.34 cM (LG 20), with an average of 75.26 cM. A total of 589 shared markers (6.94%) were heterozygous in both parents, and the number of shared markers for each group ranged from six (LG 7 and LG 47) to 29 (LG 9), with an average of 11.78. A total of 607 slight segregation distortion markers (7.15%) were distributed across the linkage maps, and they mainly gathered in some LGs such as LG1, LG6, LG12, LG22, and LG50 (Table 1 and Supplemental Material, Table S1). The expected map length was calculated to be 3807.22 cM (*G*_e1_) and 3809.784 cM (*G*_e2_), and the average *G*_e_ of 3808.5 cM from these two estimation methods is taken as the expected genome length of crucian carp. The genome coverage of this consensus genetic map is then 98.8%.

**Table 1:** Summary of statistics for the consensus linkage map, female-specific map, and male-specific map of crucian carp

Linkage Group	Consensus Map							Female-Specific Map				Male-Specific Map				F:M Ratio	
	Mapped Markers	Female-Specific Markers	Male-Specific Markers	Shared Markers	Distorted Markers	Genetic Length (cM)	Marker Interval (cM)	Mapped Markers	Distorted Markers	Genetic Length (cM)	Marker Interval (cM)	Mapped Markers	Distorted Markers	Genetic Length (cM)	Marker Interval (cM)	Map Length ratio	Recombination Rate Ratio
1	185	72	104	9	40	86.012	0.465	81	23	73.046	0.902	113	19	76.238	0.675	0.958	1.449
2	187	74	101	12	13	84.78	0.453	86	9	74.402	0.865	112	4	68.646	0.613	1.084	0.902
3	180	79	88	13	9	59.365	0.33	91	9	62.601	0.688	102	5	52.268	0.512	1.198	2.66
4	193	72	104	17	7	77.119	0.4	89	3	69.408	0.78	120	5	76.537	0.638	0.907	1.037
5	236	108	119	9	14	79.266	0.336	115	12	70.974	0.617	127	2	77.126	0.607	0.92	1.502
6	325	142	165	18	41	71.661	0.22	158	39	65.037	0.412	182	5	70.877	0.389	0.918	0.924
7	166	48	112	6	15	81.564	0.491	54	10	75.841	1.404	118	6	65.469	0.555	1.158	4.115
8	171	83	80	8	1	66.045	0.386	91	1	63.417	0.697	85	0	37.254	0.438	1.702	3.334
9	241	114	98	29	9	79.169	0.329	144	4	59.244	0.411	122	7	58.94	0.483	1.005	0.893
10	223	101	109	13	11	85.396	0.383	114	9	64.729	0.568	122	2	62.688	0.514	1.033	1.943
11	139	66	64	9	13	64.123	0.461	68	1	61.925	0.911	73	13	45.025	0.617	1.375	1.822
12	118	65	42	11	20	55.264	0.468	78	7	60.677	0.778	54	15	52.452	0.971	1.157	2.175
13	230	103	108	19	8	82.736	0.36	122	6	79.948	0.655	128	2	71.358	0.557	1.12	1.148
14	239	111	115	13	5	72.021	0.301	124	2	73.23	0.591	128	3	68.096	0.532	1.075	1.529
15	152	74	62	16	8	44.947	0.296	90	8	73.738	0.819	86	0	65.858	0.766	1.12	1.587
16	138	56	74	8	12	81.189	0.588	61	8	64.905	1.064	82	5	70.366	0.858	0.922	2
17	197	100	80	17	4	77.453	0.393	111	0	61.138	0.551	97	4	75.718	0.781	0.807	0.937
18	168	81	74	13	8	83.216	0.495	94	3	73.719	0.784	87	5	81.538	0.937	0.904	1.401
19	139	64	65	10	5	63.087	0.454	74	3	68.459	0.925	77	4	72.514	0.942	0.944	1.201
20	160	74	77	9	17	100.34	0.627	82	3	84.895	1.035	86	13	65.021	0.756	1.306	0.656
21	153	73	71	9	14	97.775	0.639	82	11	74.23	0.905	80	3	73.461	0.918	1.01	2.085
22	106	45	54	7	23	73.254	0.691	49	11	73.902	1.508	61	15	57.965	0.95	1.275	2.02
23	142	80	53	9	5	67.343	0.474	89	2	59.198	0.665	62	4	60.652	0.978	0.976	1.297
24	143	60	63	20	3	54.811	0.383	70	2	59.295	0.847	83	1	68.606	0.827	0.864	1.051
25	150	78	61	11	12	68.788	0.459	89	3	73.513	0.826	77	9	73.631	0.956	0.998	2.157
26	182	86	86	10	11	85.182	0.468	96	5	71.992	0.75	96	6	88.668	0.924	0.812	3.644
27	183	65	99	19	10	76.556	0.418	84	10	65.104	0.775	118	0	66.393	0.563	0.981	1.26
28	175	81	84	10	15	91.374	0.522	91	10	70.312	0.773	93	6	70.781	0.761	0.993	0.956
29	127	67	51	9	17	92.633	0.729	76	0	80.124	1.054	60	17	87.018	1.45	0.921	2.156
30	166	68	82	16	11	59.579	0.359	84	8	76.966	0.916	100	3	75.772	0.758	1.016	1.838
31	128	53	68	7	11	88.488	0.691	60	9	63.004	1.05	75	2	68.527	0.914	0.919	2.098
32	172	75	88	9	17	79.599	0.463	84	17	64.158	0.764	97	0	63.495	0.655	1.01	3.485
33	130	81	40	9	19	72.02	0.554	90	6	76.433	0.849	49	14	70.37	1.436	1.086	1.318
34	159	64	84	11	7	72.538	0.456	75	4	67.778	0.904	94	3	53.714	0.571	1.262	1.399
35	135	57	70	8	17	75.293	0.558	65	10	73.712	1.134	77	7	61.64	0.801	1.196	1.242
36	161	81	66	14	5	76.374	0.474	92	4	65.641	0.713	80	1	60.095	0.751	1.092	1.321
37	193	98	84	11	6	76.941	0.399	109	4	83.944	0.77	95	2	73.089	0.769	1.149	3.22
38	167	83	67	17	6	78.142	0.468	100	4	68.303	0.683	84	2	68.659	0.817	0.995	1.641
39	155	64	78	13	10	75.382	0.486	70	4	63.262	0.904	91	7	69.754	0.767	0.907	1.172
40	156	71	73	12	18	73.448	0.471	83	3	71.437	0.861	83	16	62.339	0.751	1.146	1.282
41	165	83	73	9	6	69.916	0.424	92	6	67.234	0.731	81	0	59.7	0.737	1.126	1.648
42	143	55	75	13	9	72.483	0.507	68	7	74.443	1.095	88	4	67.966	0.772	1.095	1.444
43	160	76	75	9	5	72.5	0.453	85	4	68.6	0.807	83	1	39.898	0.481	1.719	2.742
44	331	154	162	15	7	80.236	0.242	169	3	69.803	0.413	177	5	79.798	0.451	0.875	1.058
45	179	77	94	8	10	81.51	0.455	85	7	71.925	0.846	102	3	74.455	0.73	0.966	1.515
46	155	64	80	11	7	74.091	0.478	75	4	71.991	0.96	90	4	70.867	0.787	1.016	0.903
47	123	54	63	6	15	79.269	0.644	60	8	71.565	1.193	69	7	66.677	0.966	1.073	13.759
48	139	62	67	10	13	74.401	0.535	72	8	78.436	1.089	77	5	63.714	0.827	1.231	1.71
49	116	60	45	11	15	60.238	0.519	72	3	79.882	1.109	57	13	66.548	1.168	1.2	9.166
50	106	61	37	8	23	67.962	0.641	67	12	62.9	0.939	45	13	68.084	1.513	0.924	2.561
Total	8487	3863	4034	590	607	3762.879	0.443	4410	349	3500.42	0.794	4625	292	3346.325	0.724	1.046	2.127

The female map comprised 4410 SNPs and spanned 3500.42 cM, with an average marker interval of 0.79 cM (Table 1 and Figure S1). The number of markers per group varied from 49 (LG 22) to 169 (LG 44), with an average of 88.20, and the length ranged from 59.20 cM (LG 23) to 84.90 cM (LG 20), with an average of 70.01 cM. While the male map consisted of 4625 SNPs, and spanned 3346.33 cM with an average marker interval of 0.72 cM (Table 1 and Figure S2). The number of markers in each group varied from 45 (LG 50) to 182 (LG 44) with an average of 92.50, and the lengths of the LGs ranged from 37.25 cM (LG 8) to 83.75 cM (LG 26), with an average of 66.93 cM. The female and male maps have 349 (7.9%) and 292 (6.3%) distorted markers, respectively.

Marker orders are largely conserved between female map and male map, although some LGs showed minor intrachromosomal rearrangements (Figure S3). The average recombination ratio of female-to-male is 2.13:1 (Table 1). Of the 50 LGs, 43 showed higher recombination rate in males than females, and, in contrast, seven LGs showed lower recombination rate in males than females. Interestingly, significant differences in recombination ratios between the female and male maps were observed in LG47 (13.76:1) and LG49 (9.17:1) (Table 1 and Figure S3).

### Comparative genome analysis

**Figure 2:**
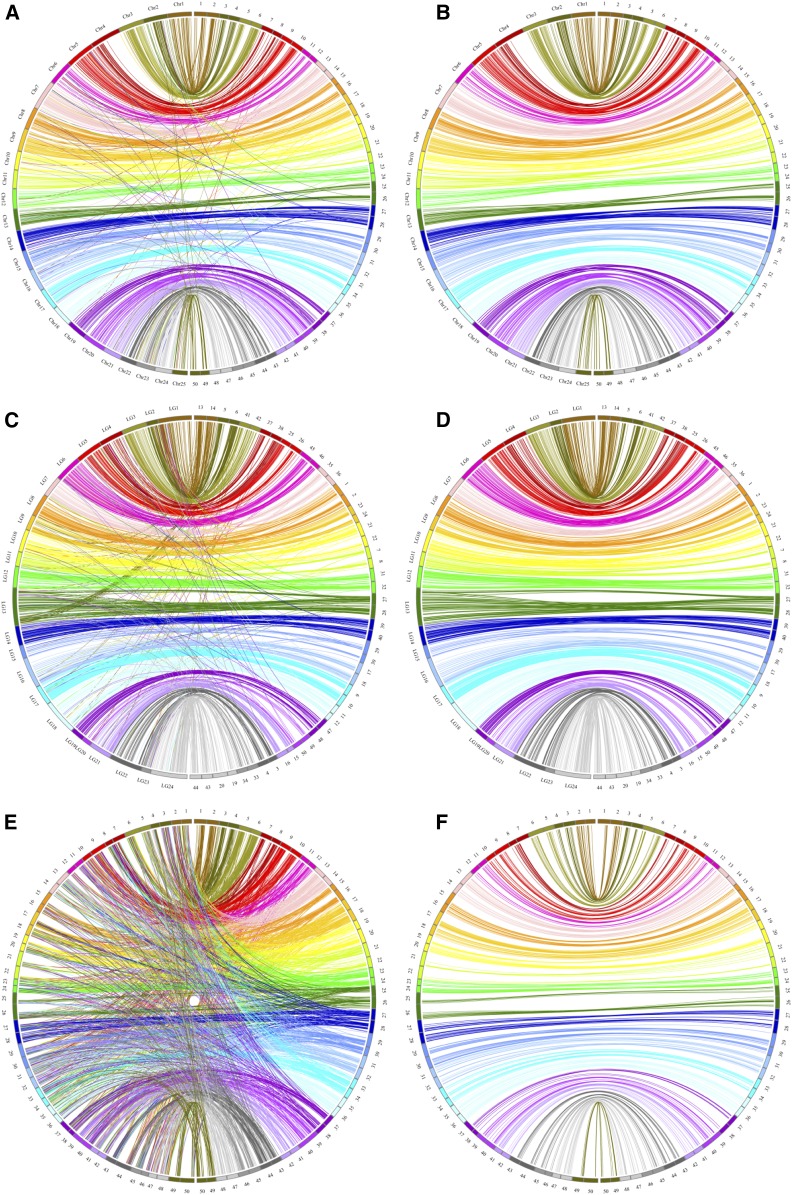
Circos diagram representing syntenic relationships between crucian carp and zebrafish (A and B), grass carp (C and D), and common carp (E and F). (B, D, and F) show only 1:2, 1:4 or 1:1 perfect orthologous pairs.

**Figure 3:**
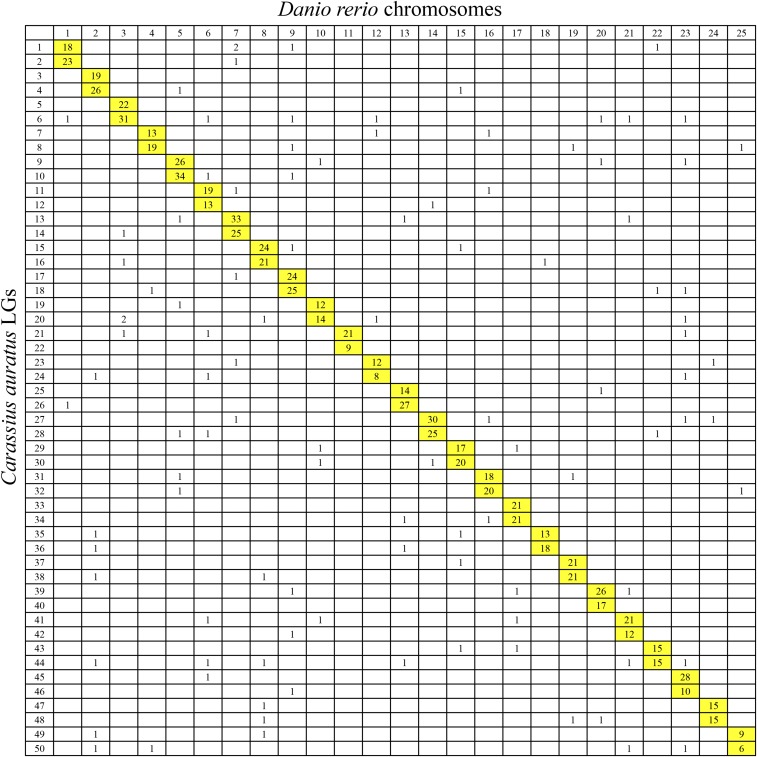
Genomic synteny visualized using Oxford grids between crucian carp linkage groups and zebrafish chromosomes. The numbers in each cell represent number of homologous loci between crucian carp consensus linkage groups and zebrafish chromosomes. Homologous linkage groups and chromosomes are highlighted in yellow.

**Figure 4:**
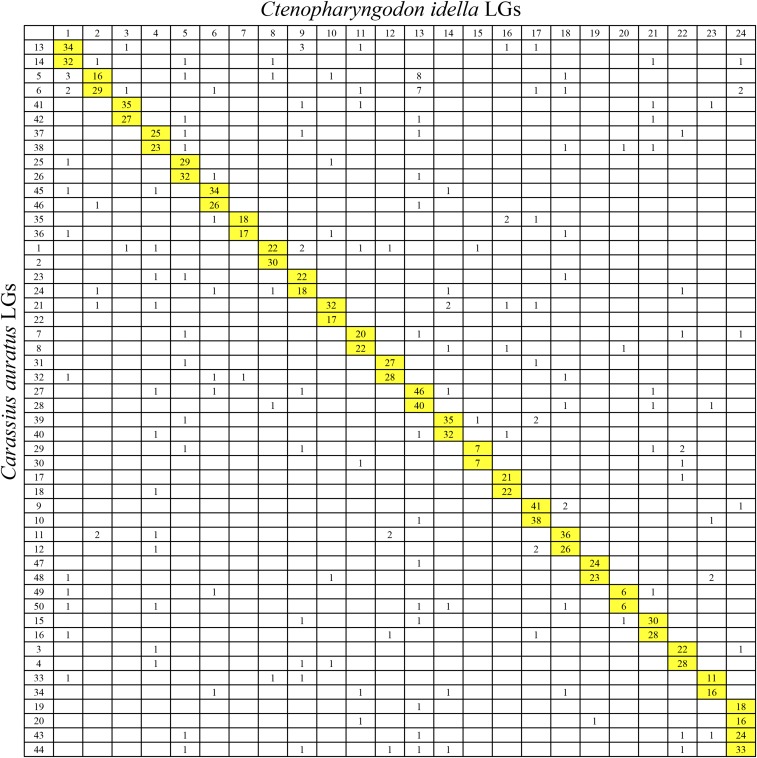
Genomic synteny between crucian carp linkage groups and grass carp linkage groups visualized using Oxford grids. The numbers in each cell represent number of homologous loci between crucian carp consensus linkage groups and grass carp linkage groups. Homologous linkage groups are highlighted in yellow.

**Figure 5:**
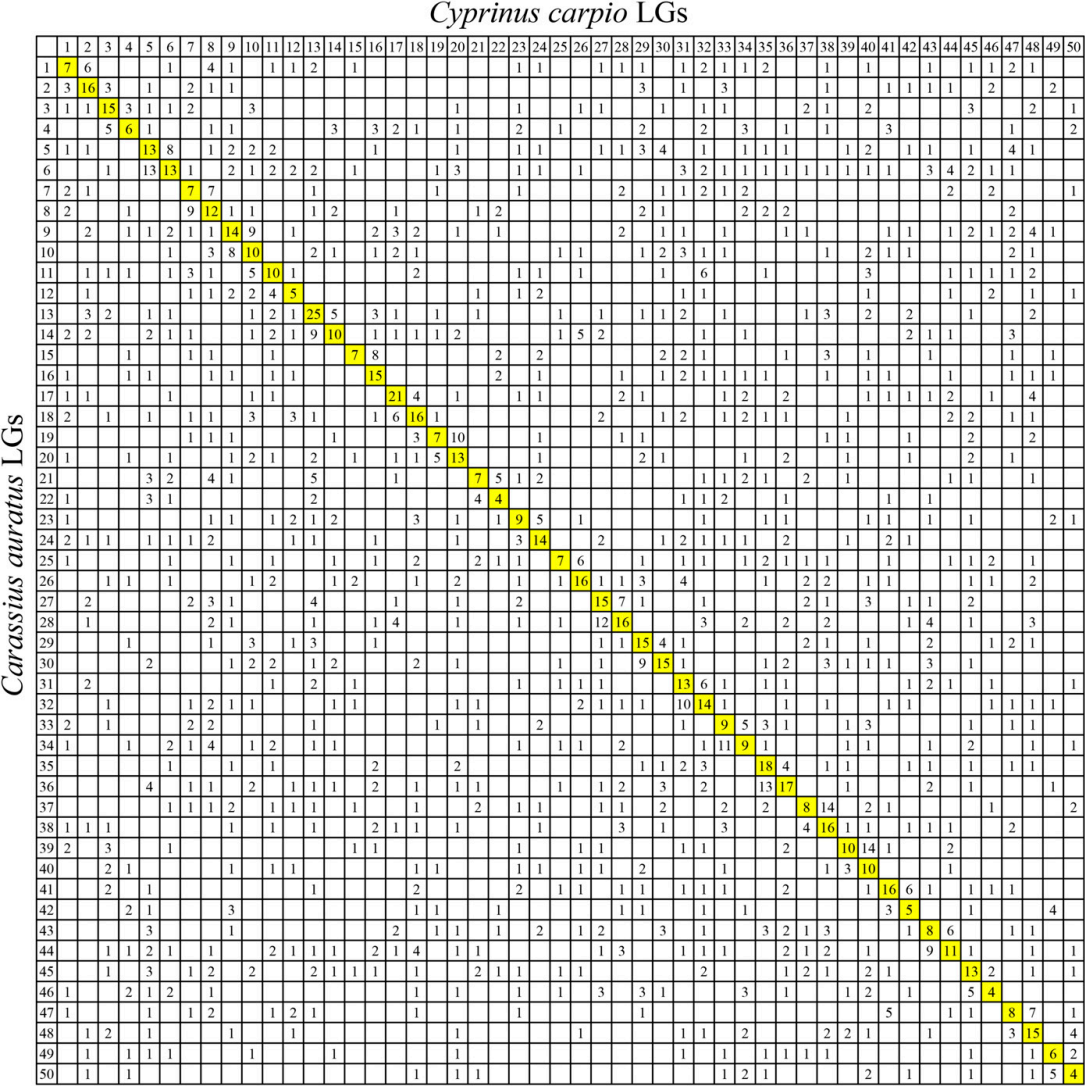
Genomic synteny between crucian carp linkage groups and common carp chromosomes visualized using Oxford grids. The numbers in each cell represent number of homologous loci between crucian carp consensus linkage groups and common carp chromosomes. Homologous linkage groups and chromosomes are highlighted in yellow.

Comparative genomic analysis was performed between crucian carp LGs and zebrafish, grass carp, and common carp chromosomes. For 5734 extended sequences of SNP markers, a total of 1072 markers was uniquely aligned to the genome of zebrafish. The results showed that two LGs of crucian carp were homologous to one particular chromosome of zebrafish, suggesting a clear 2:1 relationship of crucian carp LGs and zebrafish chromosomes (Figure 2A and Figure 3). Of the 1072 orthologous pairs, 966 pairs (90.1%) were located into the conserved syntenic blocks, revealing highly conserved synteny between crucian carp and zebrafish genome (Figure 2B). Of note, 49% regions (37–75 Mb) on chromosome 4 of zebrafish had no homologous segments in crucian carp (Figure 2, A and B). A total of 1441 orthologous pairs between crucian carp and grass carp were identified, with 1267 (87.9%) mapped on those paired collinear blocks (Figure 2C). Comparison analysis showed a 2:1 synteny between crucian carp and grass carp LGs, except for LG 24 in grass carp, which was aligned to four LGs in crucian carp (LG19, LG20, LG43, and LG44) (Figure 2D and Figure 4). The comparative genomics results show that intensive chromosomal rearrangements were present between crucian carp and common carp (Figure 2E and Figure 5). Of the 2201 markers uniquely anchored to the chromosomes of common carp, only 574 (26.1%) 1:1 orthologous pairs were identified (Figure 2F).

### QTL for body weight

**Figure 6:**
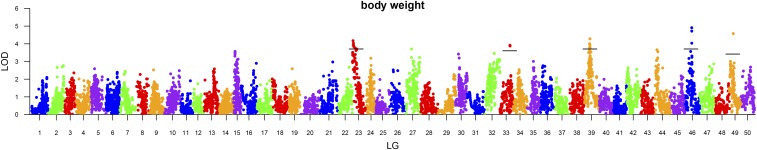
A genome scan of LOD profiles for body weight. The solid lines indicate the chromosome-wide significance thresholds.

**Figure 7:**
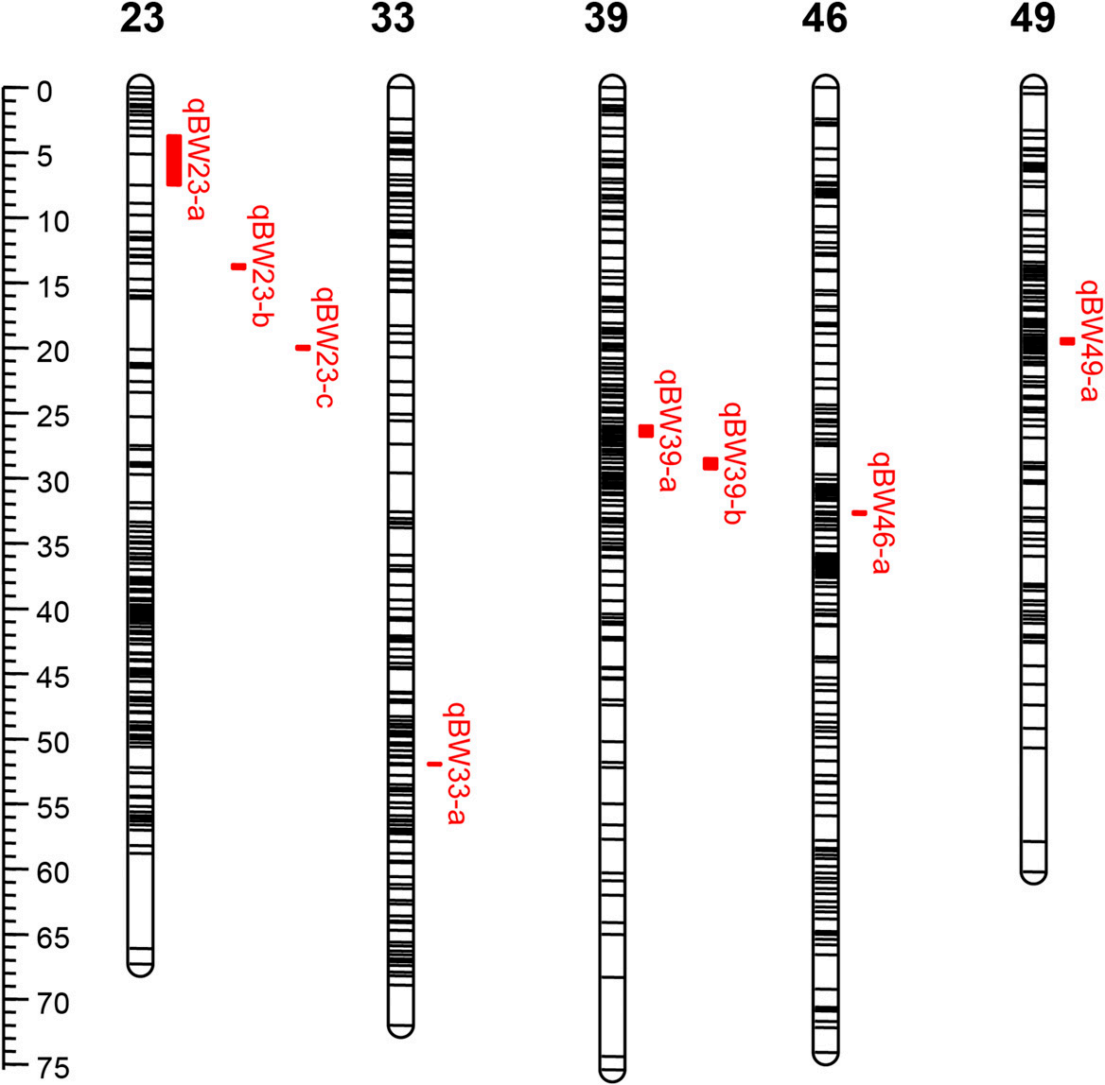
The distribution of eight QTL for body weight on five genetic linkage groups of crucian carp.

QTL fine mapping based on above high-density genetic linkage map showed that eight chromosome-wide QTL associated with body weight were identified. These QTL distribute on five LGs (LG23, LG33, LG39, LG46, and LG49), with LOD scores from 3.71 to 4.91, and the phenotypic variance explained (PVE) ranging from 10.1 to 13.2% (Figure 6, Figure 7, and Table 2). The highest PVE value was detected for qBW46-a on LG46 with the LG positions of 32.579–32.808 cM (Table 2). All QTL intervals in this study are <1 cM except qBW23-a on LG23 (3.765 cM), with an overall average 0.87 cM for eight QTL intervals. Each QTL interval harbors 1–3 SNP markers (Table 2).

**Table 2:** Summary statistics of the QTL for body weight and uncovered candidate genes in crucian carp

QTL name	LG	Position (cM)	No. of SNPs	LOD	Chromosome -Wide Threshold	PVE (%)	Nearest Marker	Candidate Genes
qBW23-a	23	3.694–7.459	3	4.17	3.6	11.3	ref-33789_15	Epidermal growth factor-like domain
qBW23-b	23	13.039–13.467	1	3.77	3.6	10.3	ref-102483_29	Immunoglobulin-like
qBW23-c	23	19.228–20.054	1	3.71	3.6	10.1	ref-28586	
qBW33-a	33	51.928–52.005	2	3.92	3.6	10.7	ref-68343_4	Zinc finger, C2H2
qBW39-a	39	26.023–26.755	2	4	3.7	10.9	ref-123717	
qBW39-b	39	28.471–29.264	3	4.28	3.7	11.6	ref-96199	Transforming growth factor β
qBW46-a	46	32.579–32.808	3	4.91	3.7	13.2	ref-38422_10	Protein kinase, ATP binding site
qBW49-a	49	19.534–19.65	1	4.58	3.4	12.3	ref-103659	

### Potential candidate genes

BLASTN searches of QTL-associated SNP sequences against the genome sequences of crucian carp and common carp gave five potential candidate genes (Table 2). Markers in three QTL (qBW23-c, qBW39-a, and qBW49-a) failed to reveal any potential candidate genes because they were unable to align to any of the two genomes. Among those annotated genes with known biological functions (Table 2), epidermal growth factor-like domain (EGF-like domain) and transforming growth factor β (TGF-β) are two representatives.

## Discussion

With the development of high-throughput sequencing technology and the advancement of GBS methods, constructing well-defined genetic linkage maps using thousands of SNP markers is now available in many nonmodel organisms, including aquaculture species. High-density SNP linkage maps have been constructed using 2b-RAD sequencing in several aquaculture animals, including *Chlamys farreri* (3806 SNPs) (Jiao *et al.* 2014), pearl oyster (3117 SNPs) (Shi *et al.* 2014), Chinese mitten crab (10,358 SNPs) (Cui *et al.* 2015), sea cucumber (7839 SNPs) (Tian *et al.* 2015), bighead carp (3121 SNPs) (Fu *et al.* 2016), and gilthead sea bream (Palaiokostas *et al.* 2016) (12,085 SNPs). The number of markers on a linkage map is usually determined by the choice of restriction enzymes, the number of restriction enzymes, the total number of enzyme cut sites, and the polymorphism rate across the genome (Andrews *et al.* 2016). In this work, taking advantage of 2b-RAD technology, we constructed a high-density linkage map containing 8487 SNP markers with a resolution of 0.44 cM for crucian carp. This robust genetic linkage map will contribute to a better understanding of the genome structure, function, and evolution of crucian carp. In addition, the linkage map will be highly valuable to fine mapping for more complex traits and chromosome assembly of WGS in crucian carp.

Sex differences in recombination rates have been reported in a number of teleosts (Zhu *et al.* 2014). In many fishes, females usually have a higher recombination frequency than males (Liu *et al.* 2013). Recombination ratio differences between female and male have been observed in zebrafish (2.74:1) (Singer *et al.* 2002), rainbow trout (1.68:1 and 3.25:1) (Rexroad *et al.* 2008; Sakamoto *et al.* 2000), Atlantic halibut (1.89–2.53:1) (Reid *et al.* 2007), turbot (1.6:1) (Bouza *et al.* 2007), barramundi (2.06:1) (Wang *et al.* 2007), and grass carp (2.03:1) (Xia *et al.* 2010). In this study, the average female to male recombination ratio of crucian carp was 2.13:1, which was similar to many other fish species. In the majority of female LGs, the recombination ratios were obviously higher than those of male LGs. However, significant male-biased recombination suppression was observed in LG47 and LG49. This is a very interesting phenomenon worthy of attention. Previous studies have suggested many factors, such as sex chromosomes, regions around centromeres and/or telomeres, large areas of repetitive DNA, and heterochromatin could influence recombination rate (Ninwichian *et al.* 2012; Jones *et al.* 2013). Recombination suppression between sex chromosomes is a common phenomenon in vertebrates, and is important in maintaining the stability of the sex-determining regions and leads to the degeneration of Y or W chromosomes (Charlesworth *et al.* 2005; Bergero and Charlesworth 2009). In three spine sticklebacks and medaka, recombination in the sex determination region is reduced in the male linkage map relative to the female linkage map (Peichel *et al.* 2004; Roesti *et al.* 2013; Matsuda *et al.* 1999; Naruse *et al.* 2000). It was reported that crucian carp has an XX/XY sex chromosome system (Yamamoto 1974), and has a pair of heteromorphic chromosomes that were taken as X and Y chromosomes (Ruiguang 1982). In our study, significant male-biased recombination suppression on LG47 and LG49 may suggest that these two LGs are potential sex chromosomes of the crucian carp genome. Further studies are required to confirm this hypothesis, and elucidate the genetic mechanism for sex determination in crucian carp and other cyprinid fishes.

A high-density genetic map constructed with sequence-based markers makes it possible for genome evolution studies in nonmodel species (Z. Wang *et al.* 2015; H. Liu *et al.* 2016). In this study, a detailed syntenic relationship was established between crucian carp LGs and zebrafish, grass carp, and common carp genomes via genetic maps and assembled genomes. In zebrafish, all chromosomal regions were covered by homologous loci of crucian carp except for half of Chromosome 4. The annotation information of zebrafish reference genome sequence indicated that this unique region harbored high gene duplication, high density of small nuclear RNAs (snRNAs), and may be related to sex chromosomes (Howe *et al.* 2013). This unique region presented obscure synteny with human, mouse, chicken, and common carp genomes (Howe *et al.* 2013; Xu *et al.* 2014), and our results were in accordance with those reports. The results of comparative mapping with zebrafish genome demonstrated a high level integrity of our linkage map. The conserved synteny with a clear 2:1 relationship between crucian carp LGs and zebrafish chromosomes was similar to that observed between common carp and goldfish (Peng *et al.* 2016; Xu *et al.* 2014; Zhang *et al.* 2013; Zhao *et al.* 2013; Kuang *et al.* 2016). In addition, a number of minor chromosome rearrangements were detected between crucian carp and zebrafish, which was similar to that reported in common carp (Xu *et al.* 2014). These findings suggested that crucian carp, similar to common carp, had undergone the 4R-WGD. Similarly, a clear 2:1 relationship was also observed between LGs of crucian carp and grass carp, except for LG24 of grass carp, which showed synteny to LG19, LG20, LG43, and LG44 of crucian carp. Previous studies indicated that LG24 of grass carp was orthologous to chromosome 10 and chromosome 22 of zebrafish (Z. Wang *et al.* 2015), so, after the 4R-WGD, grass carp LG24 is syntenic to four LGs of crucian carp.

Comparative analysis between crucian carp LGs, and common carp chromosomes demonstrated a 1:1 relationship, but with extensive chromosomal rearrangements. Crucian carp and common carp are two closely related species in the Cyprinidae, as they have the same number of chromosomes (2*n* = 100) and can produce hybrid offspring (Hulata 1995; Liu *et al.* 2004; S.J. Liu *et al.* 2001, 2016). Previous studies indicated that the common ancestor of crucian carp and common carp experienced the 4R-WGD ∼10.9–13.2 MYA, and the speciation occurred ranging from 8.1 to 12.9 MYA (Yuan *et al.* 2010; David *et al.* 2003; Chistiakov and Voronova 2009). A recent study confirmed that the 4R-WGD happened 8.2 MYA in common carp (Xu *et al.* 2014). Therefore, it was believed that crucian carp and common carp diverged from each other after the 4R-WGD event (Somamoto *et al.* 2005; Chistiakov and Voronova 2009). Then, the duplicated genes faced different destinations, such as nonfunctionalization, subfunctionalization, neofunctionalization, and gene dosage effects, which is very important for biological evolution, adaptation, speciation, and diversification (Glasauer and Neuhauss 2014; Lien *et al.* 2016). In this case, it can be speculated that chromosome structural difference between crucian carp and common carp could have occurred since they evolved in different directions. A similar genome structure has been seen in salmonids, in which common ancestor undergone the 4R-WGD 80 MYA, and then the genome experienced extensive chromosomal rearrangements (Berthelot *et al.* 2014; Lien *et al.* 2016; Brieuc *et al.* 2014; Guyomard *et al.* 2012; Gharbi *et al.* 2006; Naish *et al.* 2013; Timusk *et al.* 2011). Furthermore, the rediploidization process followed by the 4R-WGD would also resulted in significant genome rearrangements (Lien *et al.* 2016). Therefore, large genomic reorganizations between crucian carp and common carp may be due to the independent genome evolution and rediploidization process after genome duplication.

Growth is an important trait of interest in aquaculture species, and great efforts have been devoted to promoting the growth rate of crucian carp (Liu *et al.* 2001; Gui and Zhou 2010; Zhou *et al.* 2000a,b, 2001; Liu *et al.* 2004). However, for genetic improvement of quantitative traits, traditional breeding methods have encountered some bottlenecks and problems (Mackay *et al.* 2009). QTL fine mapping and positional cloning of candidate genes may have been an efficient approach for breeding programs in aquaculture animals, especially for quantitative traits (Peng *et al.* 2016; Yu *et al.* 2015; Xiao *et al.* 2015; Z. Wang *et al.* 2015; Tian *et al.* 2015; Shao *et al.* 2015; Li *et al.* 2015; Shi *et al.* 2014; H. Liu *et al.* 2016). In the present study, the high-density linkage map allowed us to perform QTL fine mapping for body weight, and discover potential candidate genes for early growth stage in crucian carp. As a result, eight chromosome-wide QTL associated with body weight were located in five different LGs, which reflected the complexity of this polygenic trait. However, it is worth noting that the average map length of eight QTL was 0.87 cM, and this relatively narrow genomic region would facilitate further validating and positional cloning of potential major genes for growth in crucian carp. Among five potential candidate genes identified by genomic synteny analysis, EGF-like domain and TGF-beta may be most promising because their biological functions are likely associated with early growth and development in vertebrates. For example, EGF regulates cell proliferation and growth in human, which play an important role in cellular organization and membrane repair when the tissue is destroyed (Engel 1989). TGF-β modulates cell proliferation, differentiation, apoptosis, and immune regulation (Sporn *et al.* 1986). Our QTL results and uncovered candidate growth genes would lay a foundation for genetic improvement for growth in crucian carp. Nevertheless, we must recognize that our QTL results could suffer from the limitation of the use of a single family, which may have higher power to detect family-specific or rare QTL, but could also trade off the detection power of common QTL shared across families. In the future, joint multiple population analysis will be needed to detect QTL shared among multiple families with a wider scope of inference. In addition, taking into account the Beavis effect, QTL effects tend to be overestimated as sample size is relatively small, and the genetic architecture of the character highly polygenic, as is the case with body weight.

### Conclusion

In summary, a high-density linkage map of crucian carp was constructed by 2b-RAD method with 8487 SNPs (0.44 cM/marker). Comparative genomics among four cyprinid fishes (crucian carp, zebrafish, grass carp, and common carp) provides new insights into genome duplication and chromosomal rearrangements in crucian carp. Eight chromosome-wide QTL for body weight were detected at quite narrow regions of five LGs with the PVE values of 10.1–13.2%. A few potential candidate genes, *e.g.*, EGF-like domain and TGF-β, whose biological functions are likely involved in genetic regulation of early growth, were identified from those QTL intervals. Our present study provides valuable genetic and genomic resources for further studies of comparative genomics, genome evolution, chromosome assembly, and QTL fine mapping in crucian carp.

## Supplementary Material

Supplemental material is available online at www.g3journal.org/lookup/suppl/doi:10.1534/g3.117.041376/-/DC1.
