# Supplementary figures and images for: A High-Density Genetic Linkage Map and QTL Fine Mapping for Body Weight in Crucian Carp (Carassius auratus) Using 2b-RAD Sequencing
Source: G3 (Bethesda). 2017 Jun 8;7(8):2473–87. doi: 10.1534/g3.117.041376 (PMC5555455; doi:10.1534/g3.117.041376)

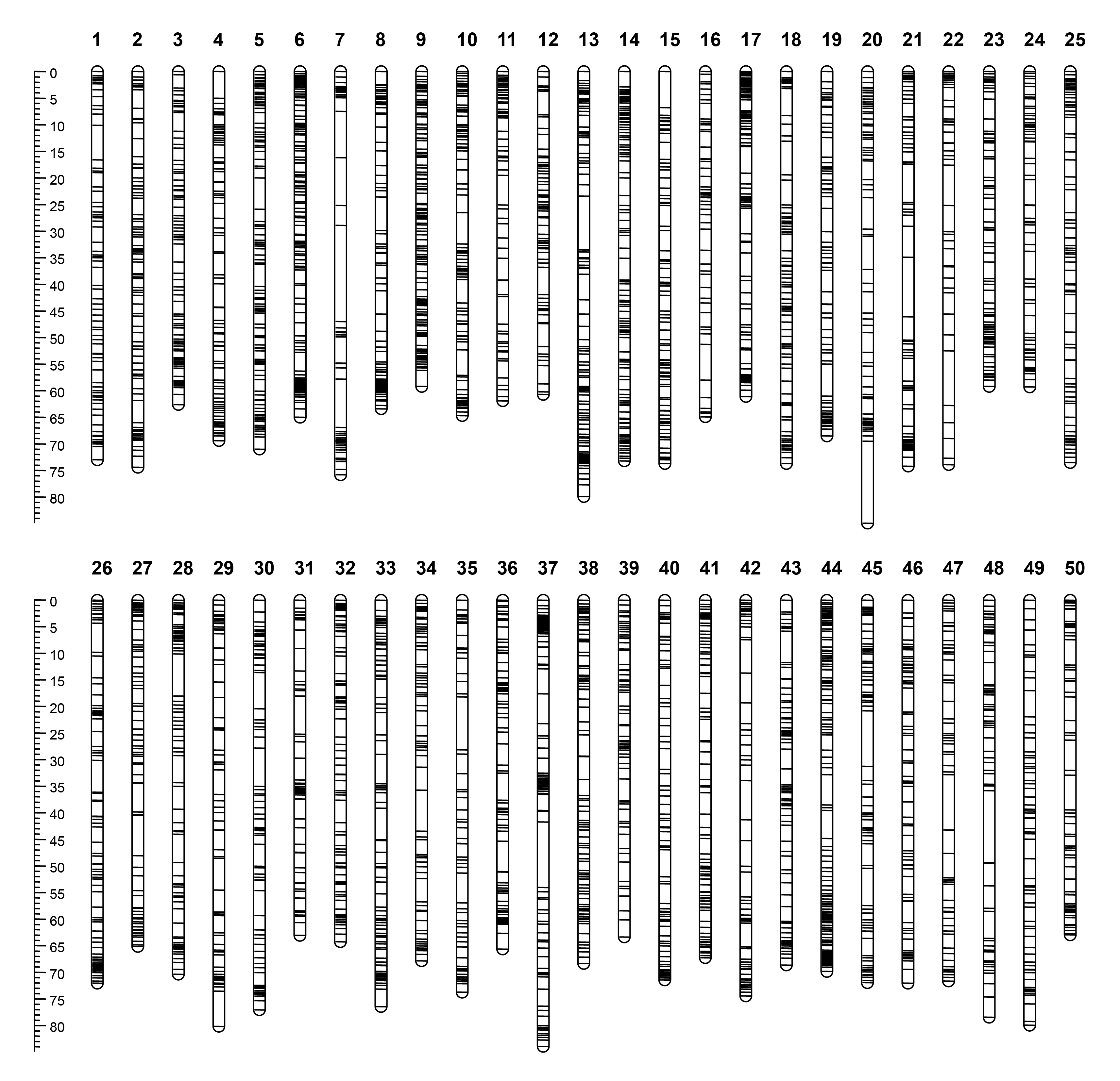

Supplement: Supplementary file 1 [file 2473FigureS1.tif]

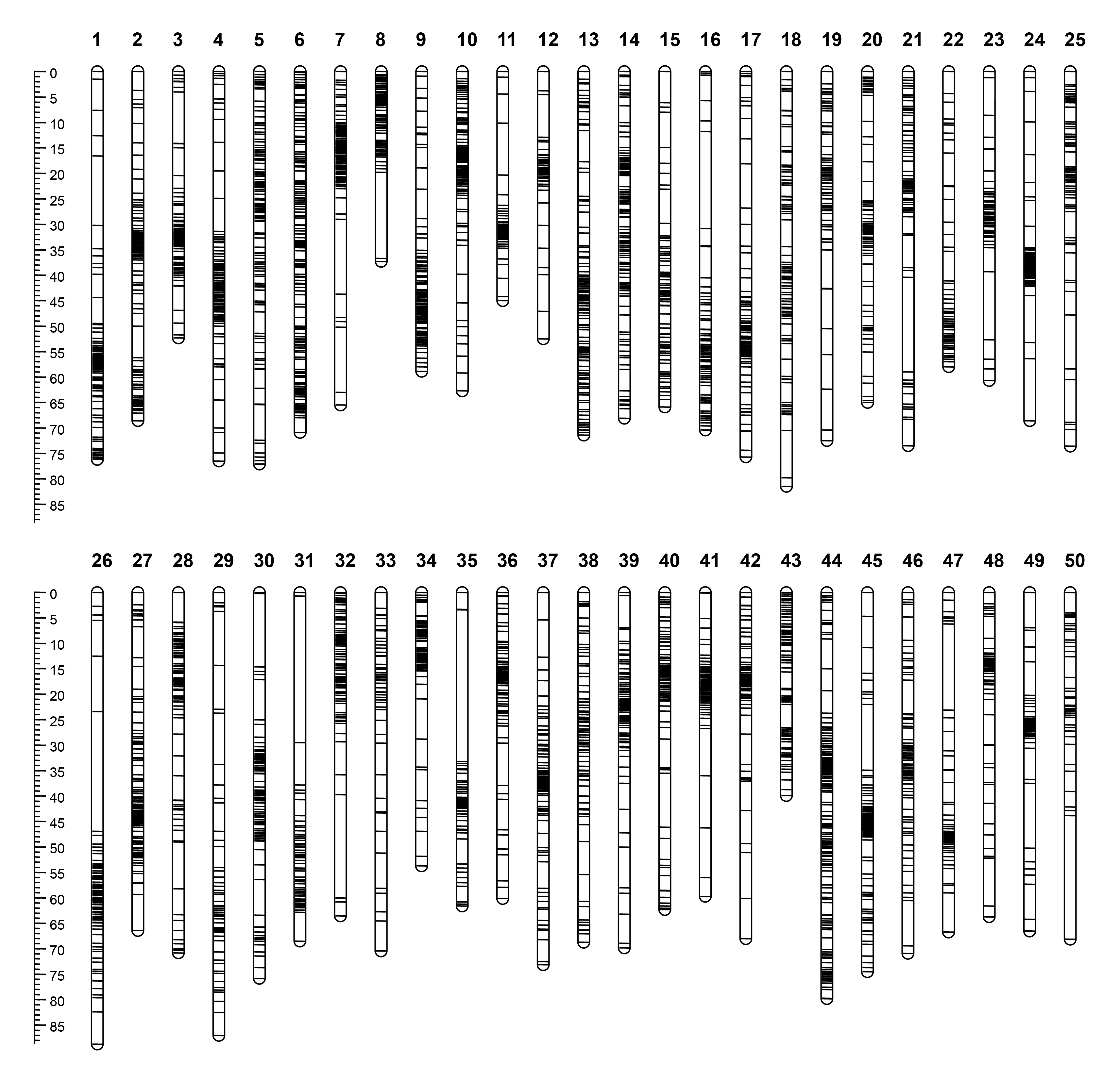

Supplement: Supplementary file 2 [file 2473FigureS2.tif]

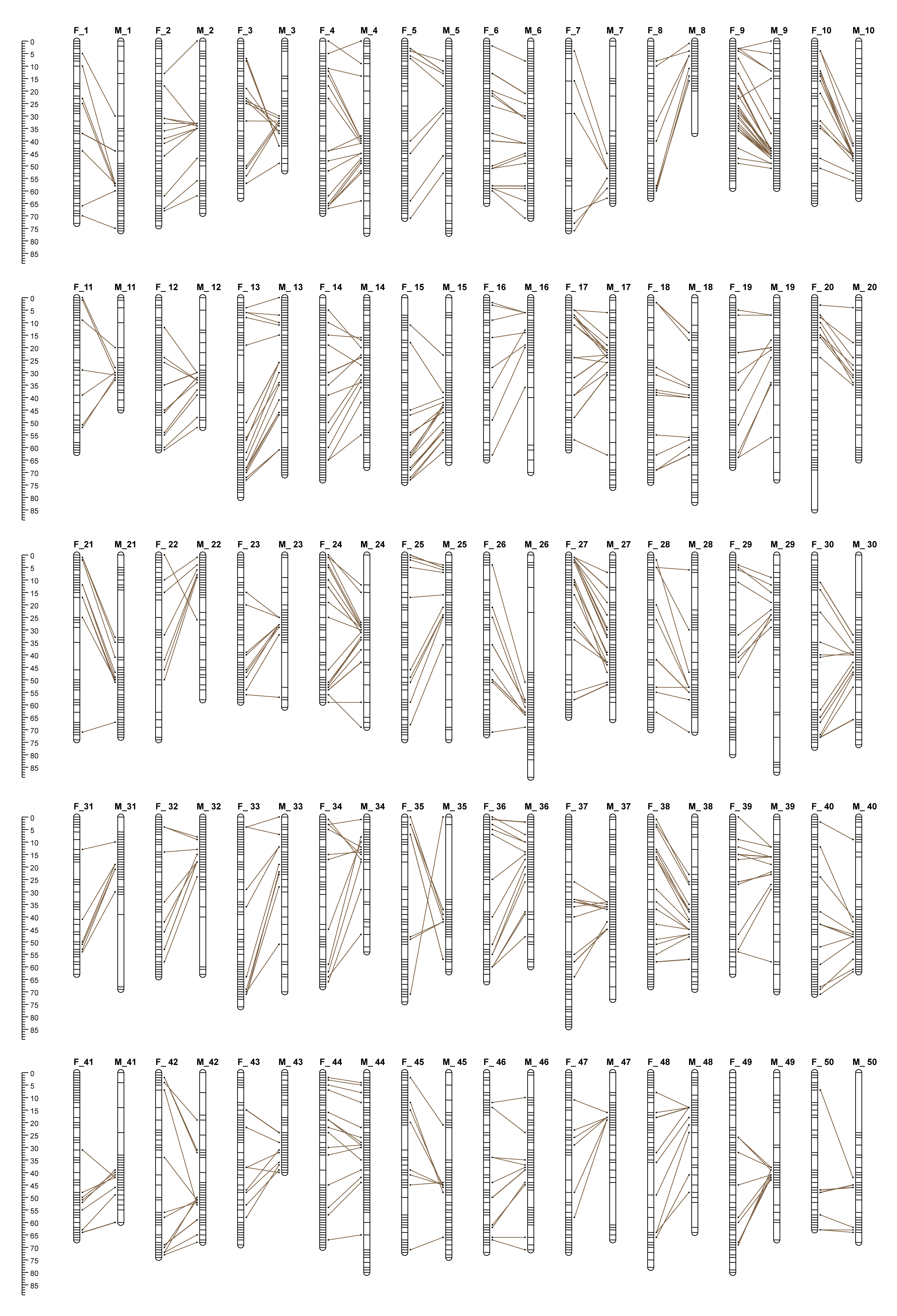

Supplement: Supplementary file 3 [file 2473FigureS3.tif]
